# Supplementary material for: Silencing of HMGA2 by siRNA Loaded Methotrexate Functionalized Polyamidoamine Dendrimer for Human Breast Cancer Cell Therapy
Source: Genes (Basel). 2021 Jul 20;12(7):1102. doi: 10.3390/genes12071102 (PMC8303903; doi:10.3390/genes12071102)
Supplement: Supplementary file 1 [file genes-12-01102-s001.zip › genes-1130283-supplementary.pdf]

Supplementary data for the ''Silencing of HMGA2 by siRNA loaded methotrexate functionalized polyamidoamine dendrimer for human breast cancer cells therapy '' manuscript.

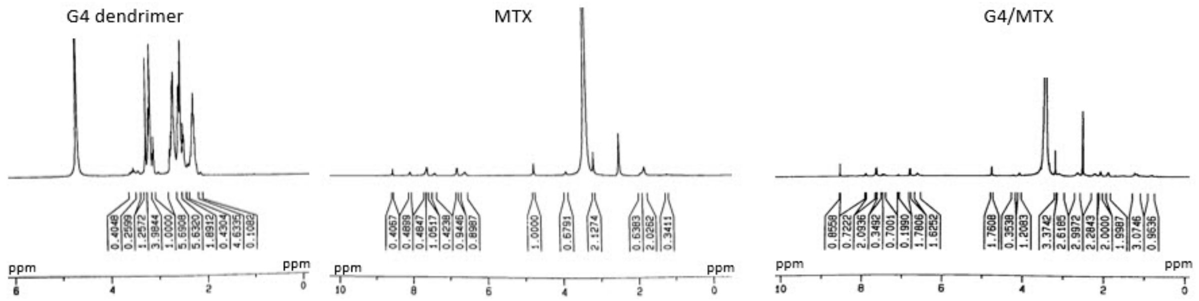

Figure S1. Representation of the NMR pack for G4, MTX and G4/MTX.
